# Interventions for the prevention of adrenal crisis in adults with primary adrenal insufficiency: a systematic review

**DOI:** 10.1530/EJE-21-1248

**Published:** 2022-05-10

**Authors:** Lisa M Shepherd, Kelly Ann Schmidtke, Jonathan M Hazlehurst, Eka Melson, Janine Dretzke, Noel Hawks, Wiebeke Arlt, Abd A Tahrani, Amelia Swift, Debbie M Carrick-Sen

**Affiliations:** 1 Diabetes & Endocrine Centre, Birmingham Heartlands Hospital, University Hospitals Birmingham NHS Foundation Trust, Birmingham, UK; 2 School of Nursing, Institute of Clinical Sciences; 3 Institute of Metabolism and Systems Research (IMSR), University of Birmingham, Birmingham, UK; 4 Centre for Endocrinology, Diabetes and Metabolism (CEDAM), Birmingham Health Partners, Birmingham, UK; 5 Medical School, University of Warwick, Coventry, UK; 6 Institute of Applied Health Research, University of Birmingham, Birmingham, UK; 7 Department of Endocrinology, Queen Elizabeth Hospital, University Hospitals Birmingham NHS Foundation Trust, Birmingham, UK; 8 Addison Disease Self-Help Group, Starling House, Bristol, UK

## Abstract

**Objective:**

The incidence of adrenal crisis (AC) remains high, particularly for people with primary adrenal insufficiency, despite the introduction of behavioural interventions. The present study aimed to identify and evaluate available evidence of interventions aiming to prevent AC in primary adrenal insufficiency.

**Design:**

This study is a systematic review of the literature and theoretical mapping.

**Methods:**

MEDLINE, MEDLINE in Process, EMBASE, ERIC, Cochrane CENTRAL, CINAHL, PsycINFO, the Health Management Information Consortium and trial registries were searched from inception to November 2021. Three reviewers independently selected studies and extracted data. Two reviewers appraised the studies for the risk of bias.

**Results:**

Seven observational or mixed methods studies were identified where interventions were designed to prevent AC in adrenal insufficiency. Patient education was the focus of all interventions and utilised the same two behaviour change techniques, ‘instruction on how to perform a behaviour’ and ‘pharmacological support’. Barrier and facilitator themes aiding or hindering the intervention included knowledge, behaviour, emotions, skills, social influences and environmental context and resources. Most studies did not measure effectiveness, and assessment of knowledge varied across studies. The study quality was moderate.

**Conclusion:**

This is an emerging field with limited studies available. Further research is required in relation to the development and assessment of different behaviour change interventions to prevent AC.

## Introduction

During acute illness or stress, the adrenal cortex produces higher amounts of the steroid hormone cortisol. Patients with adrenal insufficiency are unable to naturally produce enough cortisol and therefore are required to take daily steroid replacement therapy. These patients are advised to double or triple their dosage or to administer parental hydrocortisone during periods of acute stress, for example, during an illness, after a car accident, or before surgical intervention (1, 2). Failure to take and/or adjust their medication can lead to an adrenal crisis (AC), which can be fatal (3).

AC affects around 1 in 12 patients with primary adrenal insufficiency (PAI) each year (4). Compared to population-matched control groups, patients with PAI attend twice as many outpatient appointments and are almost five times more likely to require hospital admission (5, 6). Patients with PAI are hospitalised on average for 4.2 days vs 0.4 days for matched controls and are more likely to stay in hospital 8–10 days longer (5). Notably, patients who previously experienced an AC are at greater risk of subsequent episodes, and for every 200 incidents of AC, there will be one death (4, 7).

As managing one’s medication is behaviour based, interventions designed to change behaviour may assist patients with PAI to adopt the correct regime. Healthcare interventions that increase medication adherence tend to be education based and support the assumption that improving knowledge leads to optimal adherence. To date, such interventions have focused on increasing patients’ knowledge about their condition, how and when to take medication and the consequence of not managing their medications well (8, 9). However, previous research highlights that while patients do have the required knowledge, they do not apply it when required (10). Behavioural theory can aid the investigation of why this may be the case and can help close the knowledge–behaviour gap.

Deconstructing the interventions, to identify key components of maximum potential, is an essential step towards developing effective and acceptable future interventions (11). Utilising behavioural change models and frameworks such as the Capability, Opportunity and Motivation (COM-B) model and the Theoretical Domains Framework (TDF) provides a systematic approach to critiquing intervention components and techniques already attempted (12, 13).

There is a need to reduce the frequency and consequence of AC in people with PAI. While much is known about interventions designed to enhance medication adherence (9, 10), there is very little available evidence to inform and help people with PAI manage their medication regimens. Therefore, we undertook a systematic review to identify interventions that had been developed to prevent AC and utilised behavioural theory and frameworks to address the evidence gap.

Prior to commencing the review, we performed a search of the Cochrane database of systematic reviews, Medline (using review filter) and Epistemonikos, which yielded no results for previous systematic reviews in this topic area.

Our systematic review considered the following three research questions:

1. What interventions have been developed and evaluated to prevent AC in adult patients with PAI?
2. What is the effectiveness of the interventions?
3. What are the barriers and facilitators targeted in the interventions?

## Methods

The systematic review was prospectively registered on PROSPERO (CRD 42019137412) and is reported based on the guidelines of the preferred reporting items for systematic reviews and meta-analyses (PRISMA) statement (14) (Supplementary Table 1, see section on supplementary materials given at the end of this article).

### Search strategy

**Figure 1:**
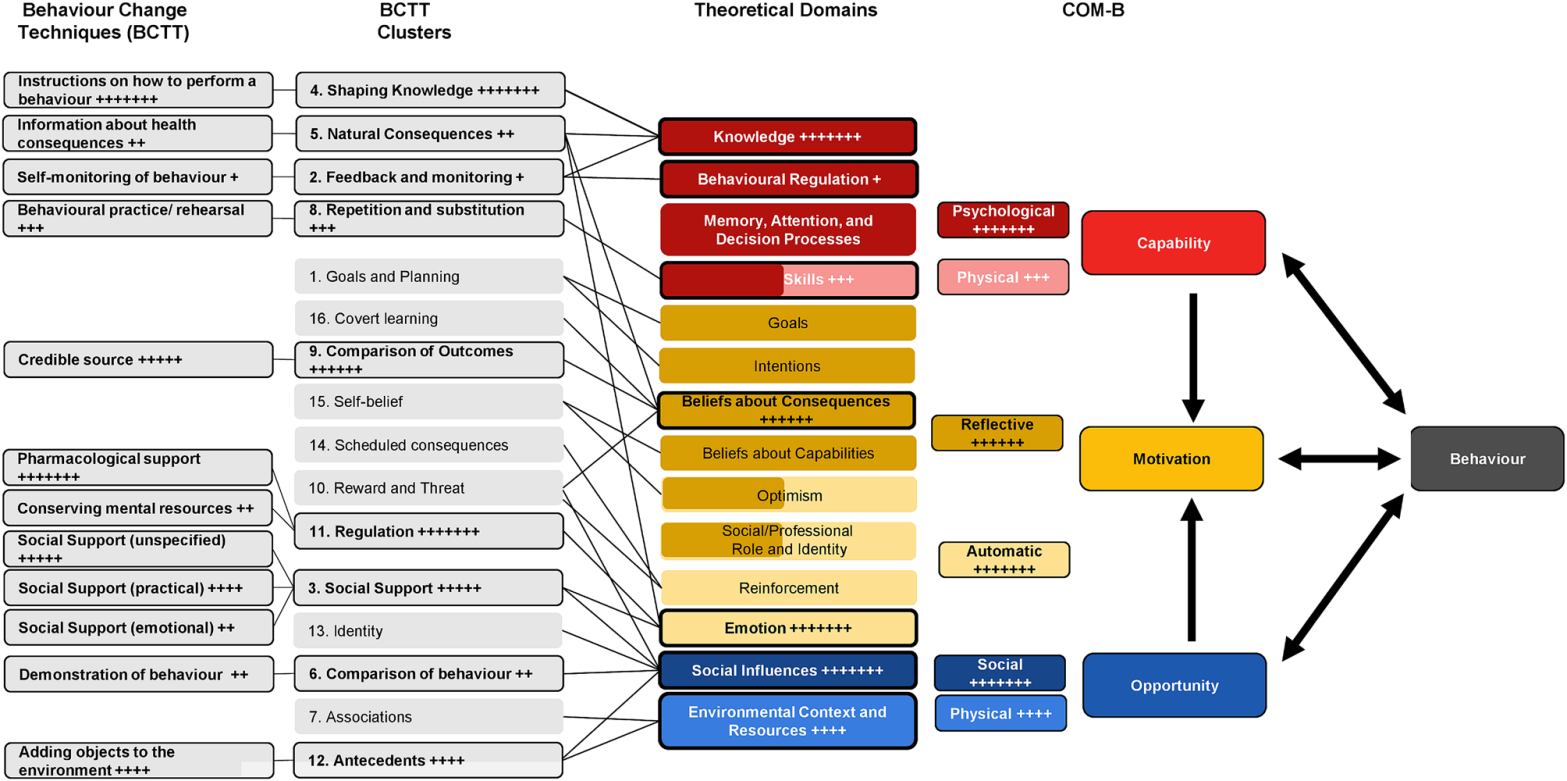
PRISMA flow diagram.

A broad search strategy was designed for MEDLINE (Supplementary Table 2) with no restrictions by publication type, study or language. The search strategy was adapted for use in different electronic bibliographical databases (15). The search terms included medical subject headings and other keywords (Supplementary Table 2). The following databases were searched from inception to November 2021: MEDLINE, MEDLINE in Process, EMBASE, ERIC, Cochrane CENTRAL for RCTs, CINAHL, PsycINFO and the Health Management Information Consortium. Trial registries were also searched including The World Health Organisation and ClinicalTrials.gov. Experts in the field were contacted, and citations of screened articles were checked to identify any further studies (Fig.1).

### Study screening and selection

Titles and abstracts were independently screened by three reviewers (LS, JH and EM) utilising Rayyan software (16). Disagreements were resolved by discussion between the reviewers. The full text of potentially relevant articles was independently screened by two researchers (LS and JH). A third reviewer (AT) was consulted to reach a consensus in case of any disagreements. The study selection process was documented with a PRISMA flow diagram.

### Study eligibility criteria

Study inclusion criteria are listed in the PICOTTS (Population, Intervention, Comparison, Outcomes, Timing, Setting and Study Design) framework to identify key characteristics (Supplementary Table 3). Papers were excluded if they contained non-empirical data and/or were an expert opinion, editorial, narrative review or conference abstracts (where the author could not provide further data on request). Only papers published in English were included, as the research team did not have financial resources to translate non-English published papers.

### Data extraction

Data were extracted using a piloted data extraction form, adapted from the Cochrane expert group (17) that is suitable for several study designs. Extracted data were independently checked by a second reviewer (JH or KS) and included study design, quality, intervention and behaviour change characteristics employed and study outcomes (i.e. incidence of AC, hospitalisation, mortality, length of stay and quality of life).

### Risk of bias assessment

The AXIS Appraisal Tool (18) was used to appraise bias in all included studies, and the Mixed Methods Appraisal Tool (MMAT) (19) was also used to appraise the van der Meij (2016) study. The risk of bias assessment was undertaken independently by LS and AS, the results were compared, and discrepancies were discussed until consensus was reached. AXIS and MMAT do not provide or encourage a numerical value for quality (18). Therefore, a descriptive summary was provided.

### Analysis/synthesis of evidence

To address the research questions, extracted data were arranged in tables and findings were reported narratively. No quantitative synthesis was possible due to the clinical and methodological heterogeneity of the studies, including interventions, outcome measures, study design and conduct.

**Figure 2:**
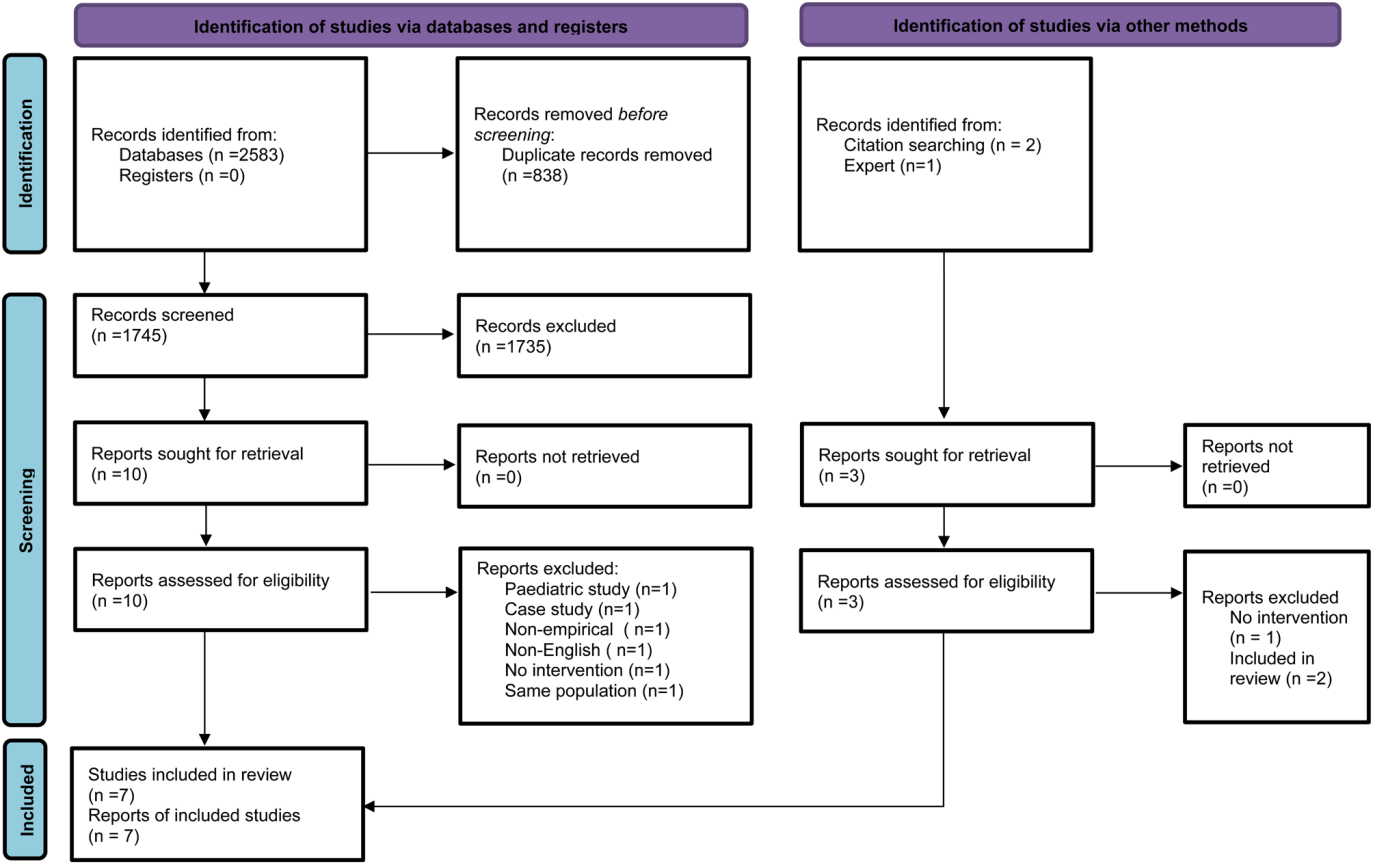
Links and frequency of identification between the BCTs, TDFS and COM-B model (adapted from Staniford and Schmidtke, 2020) + = 1 study (max *n*  = 7).

Through deductive analysis, the barriers and facilitators of the intervention were categorised into common study outcome themes. The behavioural analysis consisted of three steps. First, the key components of the interventions were mapped to the 12-point TiDIER checklist (20). Next, the behaviour change techniques (BCTs) utilised in the interventions were identified using the Behaviour Change Technique Taxonomy (BCTT) (version 1) (21). Finally, the BCTs were then mapped to link the BCTT clusters, TDF and COM-B model using the Behaviour Change Wheel components and Cane *et al.* hierarchy (12, 13, 21), see Fig. 2 for diagrammatical representation.

## Results

Seven articles were located. The PRISMA diagram describing the search is described in Fig. 1 along with reasons articles were excluded.

### Study characteristics

Out of the seven included studies, three were cross-sectional studies (22, 23, 24), three undertook cohort studies (4, 25, 26) and one utilised mixed methods (27). Questionnaires were predominately used to capture data (4, 22, 23, 24, 25). Other methods of data collection included diary (26), medical record review and semi-structured interviews (27).

The study aims predominately focused on the evaluation of patients’ knowledge (22, 27), self-management (24, 25, 26) and patients’ knowledge and self-management (23, 24). The studies (Table 1) were published between 1999 and 2020. All were conducted in European countries. Only one study focused solely on patients with PAI. The remaining studies included patients with AI and reported on these collectively, rather than separating outcome data by PAI and secondary AI. Therefore, the analysis reports collective AI. Four of the studies involved less than 100 participants and three involved more than 100 participants.

**Table 1:** Summary of characteristics of studies.

	Braatvedt *et al.* (22)	Burger-Stritt *et al.* (24)	Flemming & Kristensen (23)	Hahner *et al.* (4)	Repping-Wuts *et al.* (25)	Schöfl *et al.* (26)	Van der Meij *et al.* (27)
Location	UK	Germany	Denmark	Germany	Netherlands	Germany	Netherlands
Type of AI							
PAI	25	163	N/A	222 (includes 160 AAD)	71	34	15 (includes 7 AAD)
SAI		225	N/A	201¹	175	45	68
TAI						1	
Iatrogenic		7					
Unknown		4					
Intervention	Hydrocortisone and emergency injection education	Standardised group education information on adrenal physiology and AI GC dose adjustment during physical, or psychological stress, and AC.	Standard procedure education	Written instructions	Education group meeting	Patient recorded diaries as part of nationwide structured teaching programme	Education programme
		Emergency management and practical training of sc/im hydrocortisone injection. Equipped with an emergency card and injection set, written instructions on AI, dose adjustment and IM self-injection. Exchange of personal experiences of AI.					
Participants receiving intervention, *n*	25	526	84	423	246	80	83
Comparator	None	None	None	None	Usual care	None	None
Participants receiving comparator, *n*	Nil	Nil	Nil	Nil	44	Nil	Nil
Research objective	Determine patients with PAI knowledge of GC dose adjustment, injection supply and self-administration	Evaluate the knowledge and feelings of patients with AI in the management of adrenal emergencies following education in a standardised patient education programme.	Assess patients with PAI/SAI on HC replacement, level of information and ability to take appropriate action in cases of inter-current illness	Assess incidence, precipitating causes, potential risk factors and mortality associated with AC in patients with PAI/SAI in educated patients	Assess the self-management in patients with PAI/SAI pre and 6 months post glucocorticoid education group compared to participants who have never experienced training	Evaluate self-management of patients with PAI/SAI/TAI to enhance existing education programme	Assess educated patients with PAI/SAI knowledge of GC stress instructions and explore underlying causes and care needs in patients with insufficient knowledge
Theme focus	Patient Knowledge	Patient knowledge and self-management	Patient knowledge and self-management	Self-management	Self-management	Self-management	Patient knowledge
Design/method	Cross-sectional (one time) questionnaire-based audit	Prospective, longitudinal. multicentre questionnaire-based study	Cross-sectional (one time) questionnaire-based survey	Prospective observational, multicentre, longitudinal questionnaire (across 2 years with questionnaire every 6 months) based study.	Longitudinal questionnaire- based study; consisting of pre- and postintervention survey	Prospective, multicentre, observational diary-based study	Mixed methods study
							Quantitative methods- statistical tests using hospital records and coded interview responses (correct/ incorrect)
							Qualitative methods- content analysis of thematically coded intervention responses.
Setting	One UK endocrine unit	Four university hospital endocrine units, two endocrinology medical practices and two medical practices	One university hospital endocrine out-patient clinic	Four university hospitals	One university hospital endocrine unit	Four tertiary endocrine centres	One university hospital endocrine unit
Sample size	25	399	84	423	290	80	83
Age, years							
Mean ± s.d.	49 (18–79)				49.7 ± 15.0	52.9 ± 15.9	53.3 ± 14.4
Median (range)		55 (18–85)	59 (20–87)	50 (20–83)			
Sex ratio m:f	4:22		34:50		140:106	36:44	41:42
PAI		47:116		54:168			
AAD				37:123			
SAI		113:112		87:114			
Iatrogenic		2:5					
Unknown		1:3					
Duration of AI (years)							
Mean ± s.d.	15 (1–35)				17.0 ± 12.8; Control: 19.7 ± 11.6	14.2 ± 11.5	
Median (range)		6 (0–64)	n/a			(0.5–46)	4 (0.5–44)
PAI men				9.5(0.2–43)			
PAI women				10 (0.2–57)			
SAI men				9.5 (1–69)			
SAI women				11 (1–40)			
Study outcomes							
AC	44%	n/a	n/a	~11% (46/423)	n/a	2.5% (2/80)	25.3% (21/83)
PAI				63% 29/46			
SAI				37% 17/46 SAI			
Frequency	n/a	n/a	n/a	8.3/100 pt/yrs	n/a	2.1/100 pt/years	n/a
Hospitalisation	n/a	n/a	6% (5/84) were admitted to hospital for (AC) febrile events	14% (~59/423)	n/a	2.5% (2/80)	n/a
Deaths	n/a	n/a	n/a	~1% (4/423)	n/a	0% (0/80)	n/a
Quality of life	n/a	Prior to education 59% of patients felt they were doing ‘very well’/’well’, regarding their AI. 33% were satisfied and 7.9% felt they were doing ‘bad or very bad’. 66% of patients felt that their personal life was affected due to AI. 54% of employed patients felt AI had affected them.	n/a	n/a	n/a	n/a	n/a
Study outcomes applied to TDF domains							
Knowledge	28% (7/25) correct action, 12% totally incorrect action (3/25).	Significantly increased after education (all *P* < 0.001). Recognition of signs and symptoms of incipient AC (2.2 ± 0.7 vs 2.5 ± 0.9, *P* < 0.001) and perception of self-management was significantly better immediately after education than 6–9 months post education (2.2 ± 0.8 vs 2.6 ± 0.9. *P* < 0.001).	~54% (45/84) answered at least 4/6 hypothetical questions about acute stress correctly and either with at least one correct answer to question 7 or 8.5% (4/84) answered all questions correctly. There was a marked difference with knowledge and age. 59% (50/84) considered themselves well informed.		Comparison between baseline and follow-up in the intervention group saw an increase in the number of hypothetical questions answered correctly. Before intervention there were no significant differences between control responses to hypothetical questions about their condition. However, significantly more in the group that were to receive intervention vs responders mentioned taking action in case of flu and raised temp ≥38°C. After the intervention, the treatment group were more likely to report that they would take appropriate action after vomiting and after repeated vomiting/diarrhoea and a concerning temperature.		51.8% (43/83) were unable to answer the hypothetical questions correctly. Level of education was significantly associated with knowledge.
Behaviour	60% (15/25) had never changed their GC dose despite 80% (12/15) having the disease >16 years. 8% (2/25) could self-administer (1 did not take kit on holiday). 80% (20/22) carried steroid card/wore medical alert jewellery.		~38% (14/37) of patients who had reported at least one episode of pyrexia during past year had not increased their GC dose. ~80% (67/84) possessed a steroid card	53% (n/a) of patients who reported deterioration in health did not seek medical advice. 18% (n/a) reported no GC dose adjustment. From 78 episodes of vomiting, 12% (n/a) did not adjust their steroids, 18% (n/a) used a GC suppository, 30% (n/a) adjusted their oral GC dose and 41% responded appropriately and gave parenteral GC (total = 101% due to rounding). Patients who experienced AC during follow-up were more likely to adjust GC dose during fever (89% vs 63%) and other events requiring adjustment (78% vs 62%).		~89% (71/80) of patients experienced at least 1 day of discomfort, which required dose adjustment on 35% of discomfort days. Discomfort documented in 13.6% of all recorded days. GC dose adjustment during symptoms which might indicate GI infection only 30% doubled the dose. Several patients (number unspecified) doubled or tripled their dose even though symptom score was low	4.8% never increased their dose.
Beliefs about capabilities		Significantly fewer patients would dare to perform emergency injection at 6–9 months compared to immediately after education. Younger patients (<55 years) were more confident to self-inject compared to older patients (74, 89% vs 62, 77%) at baseline and long-term follow-up. More males were confident than females to self-inject at baseline and immediately after education (75, 95% vs 64, 86%).					91.2% (62/83) of those taught, thought themselves capable to administer emergency injection.
Emotions		Significantly more patients stated that they would dare to perform an injection after education compared to baseline (68% vs 91% vs 83% *P* < 0.001). 94% patients felt that a standardised patient education programme would improve their quality of life, this increased to 98% after education and 95% 6–9 months post education			The control group were more satisfied with the information they had received in the past than the treatment group		
Skills	40% (10/25) said never had been instructed on HC injection, of whom none had supply of parental HC.						81.9% (68/83) and/or social network knew how to administer HC injection. 18% (15/83) had never received training
Social influences	Social influences & environmental context and resources - 60% (15/25) recalled instruction of parental HC, 40% (6/15) had a supply (1 had no needles/syringes, 1 expired vial) 2 thought they would not be able to self-administer						
Environmental context and resources			~80% (67/84) possessed a steroid card.	96% (~406/423) were equipped with emergency card and 30 % (~127/423) had an emergency HC kit. Patients who had experienced AC during follow-up were more likely to be in possession of emergency kit at baseline (52% vs 26%).	After the intervention, the treatment group had more self-management tools; GC instruction leaflet, medicine passport/medical alert jewellery		97.6% (78/83) had a 100 mg vial of parental HC; however, only 86.7% (72/83) had needles and syringes. 43.3% wore medic alert identification all the time.

^1^number of SAI patients reported in Tables 1 and 2 (*n* = 201), Table 3 and abstract.

AC, adrenal crisis; AE, adrenal emergency; AAD, autoimmune Addison’s disease; GC, glucocorticoid; HC, hydrocortisone; n/a, not available; PAI, primary adrenal insufficiency; Pt/yrs, patient years; SAI, secondary adrenal insufficiency; TAI, tertiary adrenal insufficiency; TDF, theoretical domains framework.

### Risk of bias assessment

The risk of bias (RoB) utilising the AXIS and MMAT (where appropriate) critical appraisal tools is reported in Supplementary Tables 4 and 5. Due to the observational nature of these studies, confounding variables introduce potential bias that in turn limits confidence in the proposed interventions and their findings. However, two studies attempted to adjust for confounders in relation to knowledge (24, 27).

Studies recruited between 26 (22) and 423 participants (4), but up to approximately 50% did not respond to invite in one study (4) and the recruitment success rate was not reported in another (22). Across all studies, response rate was as follows: not reported (22) 87% (23), 87% (24), 46% (4), 61% (25), 80% (26) 70% (27) raising the possibility of selection bias. Also, only participants who had insufficient knowledge were invited to participate in the qualitative arm of the mixed methods study (27). Two studies took measures to address non-responders (23, 25) and the concern of non-response bias by sending a further questionnaire. Outcome variables were measured using validated tools in some studies (4, 23, 24, 25). While less relevant to hard endpoints such as death, for soft endpoints such as quality of life, it was not clear if the researchers were also responsible for delivering the intervention, or if they were blinded as assessors, potentially leading to detection bias. There was no RoB with regards to funding sources or conflicts of interest in any study, although this shows as a bias on the AXIS table.

### Behavioural change interventions developed to prevent AC outcomes

#### Intervention characteristics

Table 2 describes the intervention characteristics reported applied to the TiDIER checklist (17). The rationale for intervention development in all studies (*n* = 7) was to prevent AC (4, 22, 23, 24, 25, 26, 27). Two studies also ascribed intervention development on the recommendation of Endocrine Society guidelines (24, 26, 28). No studies included details pertaining to the use of an intervention protocol or reporting guideline, for example, TiDIER (20).

**Table 2:** Table showing intervention characteristics applied to the TiDIER reporting guidelines.

	Braatvedt *et al.* (22)	Burger-Stritt *et al.* (24)	Flemming & Kristensen (23)	Hahner *et al.* (4)	Repping-Wuts *et al.* (25)	Schöfl *et al.* (26)	Van der Meij *et al.* (27)
Intervention	Hydrocortisone and emergency injection education	Standardised group education	Standard procedure education	Written instructions	Educational group meeting	National structured teaching programme	Standardised individual education
	Parental hydrocortisone available						
Why	To adequately prepare people with adrenal insufficiency to manage their GRT during intercurrent illness	To standardise and adequately prepare people with adrenal insufficiency to manage their GRT during intercurrent illness	To adequately prepare people with adrenal insufficiency to manage their GRT during intercurrent illness	Standardise information for patients with adrenal insufficiency to manage their GRT during intercurrent illness/acute need	To adequately prepare people with adrenal insufficiency and their family/friends to manage their GRT during intercurrent illness/acute need	Identify areas of patients’ self-management during times of intercurrent illness/acute need that may require additional support	To adequately prepare people with adrenal insufficiency and their family/friends to manage their GRT during intercurrent illness/acute need
What							
Materials	Equipped with an emergency injection set	Equipped with an emergency card and injection set. Written instructions on AI, dose adjustment and IM self- injection.	Equipped with a steroid card.	Equipped with written instructions on GC adaptation.	On call endocrinologist available to contact 24 h/7 days a week.	n/a	The educational material is presented as slides, and the patient is equipped with written information to take with them after the session.
Procedures	Provided instructions on GC dose adjustment	Provided information about adrenal physiology and AI, AC, dose adjustment of the daily oral GC dose during physical or psychological stress, emergency management and self-injection of HC. Practical training for patients and relatives in preparation and administration of IM or SC emergency hydrocortisone injection.	Provided instruction and information on HC treatment and dose adjustments.	Provided instructions on GC administration and to immediately contact emergency HCP for parental HC in case of diarrhoea & vomiting.	Provided information about AI, treatment, stress-related GC dose adaption, parental administration guidance (with practical training) and how/when to contact hospital. Peer support.	A national structured teaching programme provided information about AI, dose adaptation and emergency situations. In addition, to evaluate this intervention, 100 patients were asked to complete daily diary entries about their condition. For this purpose of this project, the diary is considered part of the intervention	Provided information about AI and daily medicating, training in adjusting the dose during stress and training in injection techniques. The importance of the emergency card/jewellery was discussed and provided. Travel advice was given.
		Peer support					
Who provided	Clinical unit representatives	Endocrine nurse and endocrinologist	Trained endocrinologists	Hospital researchers as part of their study.	Nursing staff	n/a	Nurse practitioner
How	n/a	Verbal; face to face; powerpoint presentation; Group (4–10 participants per session); (patient and relative)	Verbal; face-to-face	Written instructions	Verbal; face to face; video; group (12–14 pts per meeting); (patient and guest)	n/a	Verbal; face to face; individual; slide presentation; written instructions and information; (patient and caregiver)
Where	n/a	n/a	n/a	n/a	n/a	n/a	n/a
When & how much	n/a	One 2-h session	6–12 monthly clinic review with endocrinologist	Once	One 3-h session; education group meeting	n/a	60 min session; once or twice
Tailoring	n/a	n/a	n/a	n/a	n/a	n/a	n/a
							Patients and caregivers who chose not to receive training or were not able to learn the IM injection technique did not receive the complete training.
							Patients on anti-coagulants did not receive this training due to risk of haematoma.
							All were referred to the general practitioner to ask if they could administer the injection in case of persistent vomiting, watery diarrhoea and/or decreased consciousness
Modifications	n/a	n/a	n/a	n/a	n/a	n/a	n/a
How well planned/ actual	n/a	n/a	n/a	n/a	n/a	80/100 (80%) patients returned diaries	n/a

AC, adrenal crisis; AI, adrenal insufficiency; GC, glucocorticoid; GRT, glucocorticoid replacement therapy; n/a, not available.

All interventions (*n* = 7) focused on education (4, 22, 23, 24, 25, 26, 27). The interventions were delivered in varying formats: one-to-one clinician-to-patient instruction (*n* = 2) (23, 27); group education (*n* = 2) (24, 25); patient-recorded diaries as part of a structured teaching programme (26); written information only (4); and patient instruction (format unknown) (22).

Minimal information was provided about each intervention; for example, few studies included information about the frequency of intervention, supply of emergency injection kits and emergency injection training for carers or family members. The place and timing, frequency of intervention and personnel involved in delivering the intervention were not always available. Additionally, no study reported the use of theory in the development of the intervention, which is recommended when developing an effective intervention (29), or if the development of the intervention was in collaboration with patients.

#### Effectiveness of interventions

The reporting of knowledge, frequency of AC, hospitalisation, death and quality of life were varied across studies (Table 1). Two studies assessed self-management and knowledge and reported improvement post-intervention (4, 27). But the studies did not define knowledge; that is, they did not apply a theoretical underpinning of how ‘knowledge’ should be measured. Three studies used the same technique to measure knowledge, by asking patients how they would respond to hypothetical situations with objectively right and wrong answers (23, 25, 27). Assessment of knowledge was categorised as adequate or inadequate, depending on if the participant responded as taking action or not taking action in hypothetical situations. In one study, knowledge was assessed by asking patients to identify which illnesses required dose adjustment (22). Other studies described the percentage of patients who had adjusted their medication or administered an injection during intercurrent illness (4, 24, 26).

Patients’ knowledge of medication and dose adjustment was found to be insufficient to change behaviour when needed to avoid adrenal crises in all but one study (25). Two confounding variables were reported to affect participant level of knowledge, including age (23) and education level (27). With regards to self-reported behaviour, participants that undertook emergency hydrocortisone injection training (including practical training) varied between studies from 60 (22) up to 100% (24). However, during intercurrent illness, participants admitted to not increasing their dose (4, 22, 23, 26, 27).

#### Barriers and facilitators of targeted interventions

Five studies (22, 23, 24, 25, 26) did not explicitly report barriers and facilitators that helped or hindered the application of the intervention. However, themes were identified across studies and could be categorised into five main areas: knowledge, behaviour, emotions, skills, social influences and environmental context and resources (Table 1).

Preparation and administration of the emergency injection was a barrier for some participants and often relied on the support of others to perform the task (27). The number of participants who lived with someone, 63%, was reported only by one study (23). Furthermore, although invited to do so, the number of participants who attended the education with a relative, friend and/or carer was not reported (23, 24, 27). Participants felt that they could not self-inject for several reasons: no instruction in self-injection (22), lack of confidence/reduction in confidence to inject, preparation of hydrocortisone syringe too difficult (24), no support in carrying out appropriate actions during intercurrent illness and unable to attend (or refused) emergency injection training (27). However, 41 (4) and 91% (24) of participants and/or their relatives were able to administer an emergency injection when indicated and 91% thought they would be able to administer an emergency injection if required (27). Six to nine months following training, 8% of participants felt it unlikely that they could give themselves the injection compared to immediately post-training (24).

Having the necessary equipment to perform the emergency injection is also necessary to self-administer. Two studies highlighted that participants did not have appropriate equipment, and, therefore, they would not be able to administer in times of need (4, 22). Conversely in three studies, almost all participants were in possession of one or more glucocorticoid (GC) ampoules and/or an emergency kit (24, 25, 27). In two studies, there was a discrepancy between the number in possession of parenteral GC and the number in possession of a needle and syringe (22, 27).

##### Behaviour change techniques identified in interventions

The interventions included a narrow range of BCTs (see Tables 3 and 4). The mean number of BCTs per intervention was six (3, 4, 5, 6, 7, 8, 24, 25). BCT ‘instruction on how to perform a behaviour’ and ‘pharmacological support’ were identified in all interventions and related to information and medication given to the patient to self-manage their condition appropriately (4, 22, 23, 24, 25, 26, 27). Nine out of 16 BCT clusters were utilised in the studies. BCT clusters ‘shaping knowledge’ and ‘regulation’ were applied in all studies (3, 21, 22, 23, 24, 25, 26). Only one study included ‘feedback and monitoring’ in their intervention (26), which was delivered through an evaluation of self-reported self-management diaries. Interestingly, no interventions included ‘goals and planning’, ‘association’, ‘reward and threat’, ‘identify’, ‘scheduled consequences’, ‘self-belief’ or ‘covert learning’.

**Table 3:** Frequency of identifications of BCTs across interventions aligned to theoretical domains utilising Cane *et al*. (22) grouping and COM-B components.

	References						
	(22)	(24)	(23)	(4)	(25)	(26)	(27)
Behaviour change technique							
Goals and planning							
Feedback and monitoring						Y	
Social support		Y		Y	Y	Y	Y
Shaping knowledge	Y	Y	Y	Y	Y	Y	Y
Natural consequences		Y			Y		Y
Comparison of behaviours		Y			Y		Y
Associations							
Repetition and substitution		Y			Y		Y
Comparison of outcomes	Y	Y	Y		Y	Y	Y
Reward and threat							
Regulation	Y	Y	Y	Y	Y	Y	Y
Antecedents	Y	Y	Y		Y		
Identity							
Scheduled consequences							
Self-belief							
Covert learning							
Total number							
Clusters^a^	4	8	4	3	8	5	7
Domains^b^	5	7	5	4	7	6	5
Components^c^	3	3	3	3	3	3	3

^a^Behaviour change technique cluster. ^b^Theoretical Framework Domains. ^c^COM-B; Capabilities, Opportunities, Motivation.

**Table 4:** Behaviour Change Techniques Taxonomy (BCCTv1) used in studies.

Behaviour change techniques	Braatvedt *et al.* (22)	Burger-Stritt *et al.* (24)	Flemming & Kristensen (23)	Hahner *et al.* (4)	Repping-Wuts *et al.* (25)	Schöfl *et al.* (26)	Van der Meij *et al.* (27)
1. Goals and planning							
2. Feedback and monitoring						2.3-self-monitoring of behaviour	
3. Social support		3.1-social support (unspecified)		3.1-social support (unspecified)	3.1-social support (unspecified)	3.1-social support (unspecified)	3.1-social support (unspecified)
		3.2-social support (practical)		3.2-social support (practical	3.2-social support (practical)		3.2-social support (practical)
					3.3-social support (emotional)		3.3-social support (emotional)
4. Shaping knowledge	4.1- instruction on how to perform a behaviour	4.1-instruction on how to perform a behaviour	4.1- instruction on how to perform a behaviour	4.1-instruction on how to perform a behaviour	4.1-instruction on how to perform a behaviour	4.1-instruction on how to perform a behaviour	4.1-instruction on how to perform a behaviour
5. Natural consequences		5.1-information about health consequence			5.1-information about health consequence		5.1-information about health consequence
6. Comparison of behaviour		6.1-demonstration of the behaviour			6.1-demonstration of the behaviour		6.1-demonstration of the behaviour
7. Association							
8. Repetitions and substitution		8.1-behavioural practice/ rehearsal			8.1-behavioural practice/ rehearsal		8.1-behavioural practice/ rehearsal
9. Comparison of outcomes	9.1-credible source	9.1-credible source	9.1-credible source		9.1-credible source	9.1-credible source	9.1-credible source
10. Reward and threat							
11. Regulation	11.1-pharmacological support	11.1-pharmacological support	11.1-pharmacological support	11.1-pharmacological support	11.1-pharmacological support	11.1-pharmacological support	11.1-pharmacological support
					11.3-conserving mental resources		11.3-conserving mental resources
12. Antecedents	12.5-adding objects to the environment	12.5-adding objects to the environment	12.5-adding objects to the environment		12.5-adding objects to the environment		
13. Identity							
14. Scheduled consequences							
15. Self-belief							
16. Covert learning							

##### Linking behaviour change techniques to TDF and COM-B

A diagrammatical representation of how the BCTs link to the BCT clusters, TDF and COM-B is provided in Fig. 1, and an associated tabular data is provided in Table 3. The particular techniques employed in each study are further described in Table 4. The BCTs identified in the studies are linked to seven TDF domains, ‘knowledge’, ‘behavioural regulation’, ‘skills’, ‘beliefs about consequences’, ’emotion’, ‘social influences’ and ‘environmental context and resources’ (Table 5). ‘Knowledge’, ‘beliefs about consequences’, ‘emotion’ and ‘social influences’ were utilised by all studies (*n* = 7). Noticeably, this leaves seven domains (social/professional role and identity, beliefs about capabilities, optimisim, reinforcement, intentions, goals, memory, attention and decision processes) not yet applied in interventions to prevent AC and potential areas to be investigated. Four studies included more than two TDF domains (24, 25, 26, 27). Despite the absence of several targeted domains all COM-B components ‘capability’, ‘opportunity’ and ‘motivation’ were targeted overall.

**Table 5:** Barriers and facilitators targeted in individual interventions linked to TDF domains. The table presents the number of TDF domains targeted.

TDF dDomain	References						
	(22)	(24)	(23)	(4)	(25)	(26)	(27)
Knowledge	1	≥2	1	1	≥2	1	≥2
Skills	None	1	None	None	1	None	1
Beliefs about capabilities	None	None	None	None	None	None	None
Beliefs about consequences	1	≥2	1	None	≥2	1	≥2
Reinforcement	None	None	None	None	None	None	None
Intentions	None	None	None	None	None	None	None
Goals	None	None	None	None	None	None	None
Social professional role and identity	None	None	None	None	None	None	None
Social influences	1	≥2	None	1	≥2	1	≥2
Optimism	None	None	None	None	None	None	None
Emotion	1	≥2	1	1	≥2	≥2	≥2
Environmental context and resources	1	1	1	None	1	None	None
Memory, attention and decision processes	None	None	None	None	None	None	None
Behavioural regulation	None	None	None	None	None	1	None

## Discussion

To our knowledge, this is the first systematic review to examine the types of behaviour change interventions already used to prevent AC in adult patients with PAI, their effectiveness and barriers and facilitators targeted by the intervention. We identified seven studies where interventions were designed to prevent AC in adrenal insufficiency, and the focus of all interventions was patient education. The interventions had been developed with no expressed theoretical underpinning of behaviour change and most studies did not measure effectiveness. Assessment of knowledge was not uniform across studies.

In order to increase the probability of complex interventions being effective and adopted widely, they need to be fostered carefully with all relevant stakeholders (patients, carers, health workers, etc.) and developed using a systematic theoretical basis (29, 30). Hence, interventions should be co-designed with those living with primary adrenal insufficiency (31) rather than having little influence (32). While there are a wide range of theoretical models of behaviour change, the inclusion of such models was not expressed in the included studies. Previously successful interventions to address other complex health needs have benefited from their explicit use of behaviour change models, such as diabetes and smoking cessation (33, 34). However, overly complex interventions can also lead to a lack of effect or little effect where multiple intervention components fail to address real behavioural needs (35, 36). The lack of intervention effectiveness seen in the present systematic review could therefore be related to the absence of use of a theoretical model or an insufficient application of intervention techniques to address untapped barriers to behaviour change. By unpicking the intervention components of the included studies, we have identified areas that can be specifically targeted and techniques that appear to be more favourable in the future.

While all interventions in the identified studies targeted all three COM-B components, the theoretical domains targeted varied. The most frequently targeted domains were ‘knowledge’, social influence’ and ‘emotion’. Two studies (22, 23) focused on the same BCT clusters and found that patient’s self-management was inadequate following these interventions (22, 23). However, studies that adopted comparable BCT clusters in their intervention (*n* = 8) showed improvement in knowledge at follow-up (24, 25), although knowledge and confidence reported at 6–9 was reduced (24). AC still occurred following education and incidence was reported in two studies (4, 26) demonstrating that despite patients’ knowledge increasing, this knowledge must not have been applied during times of acute need. This is supported by our teams’ findings that having good knowledge does not necessarily translate into behaviour change, as participants still experienced AC (10).

While the purpose of self-management interventions is to provide patients with the skills required to manage their condition (37), our findings indicate that adrenal crises were not avoided. These results are comparable to areas of other chronic diseases, such as heart failure and diabetes where results were also variable and suggested that a multifaceted intervention approach is required (38, 39, 40). However, our review found one study that did demonstrate increased patient knowledge and confidence when performing self-injection at baseline, but this was not sustained at 6–9 months post-intervention (24). This has also been seen in other disease areas, a meta-review of quantitative reviews looking at the effect of supported self-management interventions for people with type II diabetes mellitus demonstrated improvement in HBA1c. However, the effectiveness of the intervention was dependent on the intensity and length of programme as well as ongoing support (41). Therefore, it is important that proposed interventions are deconstructed to identify key components that work, and consideration is given to, frequency, mode and delivery of intervention.

The current systematic review also adopted a wide perspective of behavioural interventions and the barriers and facilitators targeted by the interventions, specifically around adrenal insufficiency. It has identified intervention techniques already in place, but these may not be working optimally, and other areas that can be targeted to refine the intervention. The current review also highlights current research gaps in this area and the lack of theoretical underpinning related to behavioural interventions. Future assessments of behavioural interventions to reduce AC need to include a longer duration of follow-up to ascertain that appropriate application of knowledge, regarding dose adjustment, has been applied on multiple occasions. Also, future research should include more granularities of the collective data to develop our understanding of the reasons that can lead to the inappropriate application of knowledge. Better understanding of the effect of education frequency and repeated education along with specific needs of certain populations are needed. Researchers should also consider the utilisation of a theoretical framework when developing an intervention to facilitate development in a systematic way (29, 30, 42).

### Limitations and strengths

This is the first study to systematically synthesise the literature related to interventions that prevent AC in patients who have primary adrenal insufficiency. In doing so, this review picks out the barriers and facilitators the interventions were likely to address. The review does not systematically describe the barriers and facilitators the patient experience. Another limitation is the small number of studies identified, not only in the prevention of AC in adult patients with PAI but all cause AI.

Only seven studies were identified. Additionally, the heterogenous nature of the studies’ methods and outcomes do not permit us to include a meta-analysis. While the small number of studies located may limit the reliability of our results, it also highlights an opportunity for future studies to explore a largely unexplored topic.

## Conclusion

Despite the limitations of the paucity and focus of evidence, the review informs researchers and clinicians of the need to use a comprehensive approach when developing an intervention to aid self-management to prevent AC. We found education to be the only type of behavioural change technique interventions utilised, and these interventions did not demonstrate efficacy. For interventions to be successful in the prevention of AC in patients with primary adrenal insufficiency, it is not only important to identify targeted behaviour that requires change but also to incorporate behaviour change theory, throughout both the development and implementation of interventions.

## Supplementary materials

Supplementary Material

## Declaration of interest

Co-authors Wiebke Arlt and Abd Tahrani are on the editorial board of *EJE*. Wiebke Arlt and Abd Tahrani were not involved in the review or editorial process for this paper, on which he/she is listed as an author. The other authors have nothing disclose.

## Funding

L S is an ICA CDRF Award Clinical Doctoral Research Fellow (ICA CDRF-2018-04-ST2-050) and is funded by Health Education England (HEE)/National Institute for Health Research (NIHR) for this research project. The views expressed in this publication are those of the author(s) and not necessarily those of the NIHR (Partner Name), NHS or the UK Department of Health and Social Care. K A S is currently supported by the National Institute for Health Research (NIHR) Applied Research Centre (ARC) West Midlands (NIHR200165).

## Availability of data and materials

All data generated or analysed during this study are included in this article.
