# Supplementary material for: Interventions for the prevention of adrenal crisis in adults with primary adrenal insufficiency: a systematic review
Source: Eur J Endocrinol. 2022 May 10;187(1):S1–S20. doi: 10.1530/EJE-21-1248 (PMC9175553; doi:10.1530/EJE-21-1248)
Supplement: Supplementary Material [file supplementary_material.pdf]

Supplementary files:

Supplementary files Table 1 – PRISMA 2020 checklist

Supplementary files Table 2 - Medline search strategy

Supplementary files Table 3 – PICOTTS inclusion criteria

Supplementary files Table 4 –AXIS appraisal

Supplementary files Table 5 –MMAT critical appraisal

Supplementary file Table 1 -PRISMA 2020 checklist

Section and Topic	Item #	Checklist item	Location where item is reported
**TITLE**			
Title	1	Identify the report as a systematic review.	Page 1
**ABSTRACT**			
Abstract	2	See the PRISMA 2020 for Abstracts checklist.	Page 2
**INTRODUCTION**			
Rationale	3	Describe the rationale for the review in the context of existing knowledge.	Page 3
Objectives	4	Provide an explicit statement of the objective(s) or question(s) the review addresses.	Page 4
**METHODS**			
Eligibility criteria	5	Specify the inclusion and exclusion criteria for the review and how studies were grouped for the syntheses.	Page 5 & Supplementary file Table 3
Information sources	6	Specify all databases, registers, websites, organisations, reference lists and other sources searched or consulted to identify studies. Specify the date when each source was last searched or consulted.	Page 5
Search strategy	7	Present the full search strategies for all databases, registers and websites, including any filters and limits used.	Supplementary file Table 2
Selection process	8	Specify the methods used to decide whether a study met the inclusion criteria of the review, including how many reviewers screened each record and each report retrieved, whether they worked independently, and if applicable, details of automation tools used in the process.	Page 5
Data collection process	9	Specify the methods used to collect data from reports, including how many reviewers collected data from each report, whether they worked independently, any processes for obtaining or confirming data from study investigators, and if applicable, details of automation tools used in the process.	Page 6
Data items	10a	List and define all outcomes for which data were sought. Specify whether all results that were compatible with each outcome domain in each study were sought (e.g. for all measures, time points, analyses), and if not, the methods used to decide which results to collect.	Page 6
	10b	List and define all other variables for which data were sought (e.g. participant and intervention characteristics, funding sources). Describe any assumptions made about any missing or unclear information.	Page 6
Study risk of bias assessment	11	Specify the methods used to assess risk of bias in the included studies, including details of the tool(s) used, how many reviewers assessed each study and whether they worked independently, and if applicable, details of automation tools used in the process.	Page 6
Effect measures	12	Specify for each outcome the effect measure(s) (e.g. risk ratio, mean difference) used in the synthesis or presentation of results.	NA
Synthesis methods	13a	Describe the processes used to decide which studies were eligible for each synthesis (e.g. tabulating the study intervention characteristics and comparing against the planned groups for each synthesis (item #5)).	Page 6-7

Section and Topic	Item #	Checklist item	Location where item is reported
	13b	Describe any methods required to prepare the data for presentation or synthesis, such as handling of missing summary statistics, or data conversions.	NA
	13c	Describe any methods used to tabulate or visually display results of individual studies and syntheses.	Page 6-7
	13d	Describe any methods used to synthesize results and provide a rationale for the choice(s). If meta-analysis was performed, describe the model(s), method(s) to identify the presence and extent of statistical heterogeneity, and software package(s) used.	Page 6-7
	13e	Describe any methods used to explore possible causes of heterogeneity among study results (e.g. subgroup analysis, meta-regression).	NA
	13f	Describe any sensitivity analyses conducted to assess robustness of the synthesized results.	NA
Reporting bias assessment	14	Describe any methods used to assess risk of bias due to missing results in a synthesis (arising from reporting biases).	NA
Certainty assessment	15	Describe any methods used to assess certainty (or confidence) in the body of evidence for an outcome.	NA
**RESULTS**			
Study selection	16a	Describe the results of the search and selection process, from the number of records identified in the search to the number of studies included in the review, ideally using a flow diagram.	Figure 2 – Page 42
	16b	Cite studies that might appear to meet the inclusion criteria, but which were excluded, and explain why they were excluded.	Figure 2 – Page 42
Study characteristics	17	Cite each included study and present its characteristics.	Table 1 & – Pages 28-35
Risk of bias in studies	18	Present assessments of risk of bias for each included study.	Supplementary files 4 & 5
Results of individual studies	19	For all outcomes, present, for each study: (a) summary statistics for each group (where appropriate) and (b) an effect estimate and its precision (e.g. confidence/credible interval), ideally using structured tables or plots.	Pages 28-40 Tables - 1,2,3,4,5
Results of syntheses	20a	For each synthesis, briefly summarise the characteristics and risk of bias among contributing studies.	Page 8 Supplementary files 4 & 5
	20b	Present results of all statistical syntheses conducted. If meta-analysis was done, present for each the summary estimate and its precision (e.g. confidence/credible interval) and measures of statistical heterogeneity. If comparing groups, describe the direction of the effect.	NA
	20c	Present results of all investigations of possible causes of heterogeneity among study results.	Page 7-8
	20d	Present results of all sensitivity analyses conducted to assess the robustness of the synthesized results.	NA
Reporting biases	21	Present assessments of risk of bias due to missing results (arising from reporting biases) for each synthesis assessed.	Supplementary

Section and Topic	Item #	Checklist item	Location where item is reported
			files 4 & 5 & Page 8
Certainty of evidence	22	Present assessments of certainty (or confidence) in the body of evidence for each outcome assessed.	NA
**DISCUSSION**			
Discussion	23a	Provide a general interpretation of the results in the context of other evidence.	Page 13-15
	23b	Discuss any limitations of the evidence included in the review.	Page 15-16
	23c	Discuss any limitations of the review processes used.	NA
	23d	Discuss implications of the results for practice, policy, and future research.	14-16
**OTHER INFORMATION**			
Registration and protocol	24a	Provide registration information for the review, including register name and registration number, or state that the review was not registered.	Page 2
	24b	Indicate where the review protocol can be accessed, or state that a protocol was not prepared.	Page 2
	24c	Describe and explain any amendments to information provided at registration or in the protocol.	Page 2
Support	25	Describe sources of financial or non-financial support for the review, and the role of the funders or sponsors in the review.	Page 17
Competing interests	26	Declare any competing interests of review authors.	Page 17
Availability of data, code and other materials	27	Report which of the following are publicly available and where they can be found: template data collection forms; data extracted from included studies; data used for all analyses; analytic code; any other materials used in the review.	Page 17

Supplementary File Table 2 - Medline Search Strategy

1.	exp Addisons Disease/
2.	addison* disease.ti,ab.
3.	PAI.ti,ab.
4.	(adrenal adj2 insuffic*).ti,ab.
5.	(adrenal adj2 fail*).ti,ab.
6.	1 or 2 or 3 or 4 or 5
7.	exp self management/
8.	exp Health Behavior/
9.	(behav* adj2 change).ti,ab.
10.	(Behavio* adj2 interven*).ti,ab.
11.	(Behavio* adj2 manage*).ti,ab.
12.	(Behavio* adj2 techniq*).ti,ab.
13.	(therap* adj2 intervention).ti,ab.
14.	support*.ti,ab.
15.	exp self care/
16.	exp self efficacy/
17.	Preventi* measures.ti,ab.
18.	Educat*.ti,ab.
19.	7 or 8 or 9 or 10 or 11 or 12 or 13 or 14 or 15 or 16 or 17 or 18
20.	Crisis.mp.
21.	crises.mp.
22.	stress*.mp.
23.	complication*.mp.
24.	emergenc*.mp.
25.	death*.mp.
26.	hospital*.mp.
27.	quality of life.mp. or "Quality of Life"/
28.	cost*.ti,ab.
29.	psycholog*.mp.
30.	mortalit*.mp.
31.	exp Morbidity/
32.	outpatient*.mp.
33.	AC.mp.
34.	20 or 21 or 22 or 23 or 24 or 25 or 26 or 27 or 28 or 29 or 30 or 31 or 32 or 33
35.	6 and 19 and 34

Supplementary File Table 3 - Table 3 PICOTSS inclusion criteria

*PICOTSS**	*Inclusion criteria*
Patient population	**Adults aged 18 years or older<br>Patients with PAI**<br>On GRT***
Intervention	**All behavioural change interventions**
Comparison group(s)	**Usual care or any other intervention**
Outcomes (Effectiveness)<br><br><br><br>***Outcomes (Qualitative)***	**Incidence of AC*** and/or hospitalisation<br>Length of hospitalisation<br>Mortality<br><br>Quality of life<br>Identification of behaviour barriers and facilitators in the prevention of AC**
Timing	**Any time point**
Setting	**Any setting**
Study design	**Human<br>Systematic reviews<br>All quantitative<br>Any type of mixed methods or qualitative-only research that explores the barriers and/or facilitators and/or interventions to prevent AC**

*PICOTS - Population, Intervention, Comparison, Outcomes, Timing, Setting.
**PAI – Primary Adrenal Insufficiency
**GRT – Glucocorticoid replacement therapy
***AC – Adrenal Crisis

Supplementary File Table 4 –AXIS appraisal

Study	Clear aims & objectives?	Study design appropriate for aims?	Sample size justified?	Target population clearly defined?	Sample frame representative of target population?	Selection process representative of target population?	Measures taken to address non responders?	Risk factor and outcome variables measured appropriate?	Risk factor and outcome variables measured correctly using validated instruments?	Is it clear what was used to determine statistical significance and/or precision estimates?	Were the basic data adequately described?	Does the response rate raise concerns about non response bias?	Was there information about non responders described?	Were the results internally consistent?	Were the results presented for all the analyses descried in the methods?	Discussions and conclusions justified by results?	Limitations of the study discussed?	Funding sources or conflicts of interest that may affect interpretation of results?	Ethical approval or consent of participants attained?
Braatvedt et al 1990	Y	Y	N	Y	Y	DK	N	Y	N	N	Y	N	N	Y	Y	Y	N	N	N
Burger-Stritt et al 2020	Y	Y	N	Y	Y	Y	N	Y	N	Y	Y	DK	N	Y	Y	Y	Y	N	Y
Flemming & Østergaard Kristensen 1999	Y	Y	N	Y	Y	Y	N	Y	N	DK	Y	N	N	Y	Y	Y	N	N	Y
Hahner et al 2015	Y	Y	N	Y	Y	DK	N	Y	Y	Y	Y	Y	N	Y	Y	Y	Y	N	Y
Repping-Wuts et al 3013	Y	Y	N	Y	Y	Y	N	Y	Y	Y	Y	Y	Y	Y	Y	Y	Y	N	Y
Schöfl et al 2019	Y	Y	N	Y	Y	Y	N	Y	Y	Y	Y	Y	N	Y	Y	Y	Y	N	Y
van der Meij et al 2016	Y	Y	N	Y	Y	Y	N	Y	N	Y	Y	Y	N	Y	Y	Y	Y	N	Y

Key

Green	Yes
Red	No
Yellow	Don't know

Supplementary File Table 5 –MMAT (2018) critical appraisal

Study	Clear research questions?	Do the collected data allow to address the research questions?	Is the qualitative approach appropriate to answer the research question?	Qualitative data collection methods adequate to address the research question?	Findings adequately derived from the data?	Interpretation of results sufficiently substantiated by data?	Coherence between qualitative data sources, collection, analysis and interpretation?	Is randomization appropriately performed?	Groups comparable at baseline?	Are there complete outcome data?	Are outcome assessors blinded to the intervention provided?	Did the participants adhere to the assigned intervention?	Participants representative of the target population?	Measurements appropriate regarding both the outcome and intervention (or exposure)?	Are there complete outcome data?	Confounders accounted for in the design and analysis?	During the study period, is the intervention administered (or exposure occurred) as intended?	Sampling strategy relevant to address the research question?	Sample representative of the target population?	Are the measurements appropriate?	Is the risk of nonresponse bias low?	Statistical analysis appropriate to answer the research question?	Adequate rationale for using a mixed methods design to address the research question?	Are the different components of the study effectively integrated to answer the research question?	Are the outputs of the integration of qualitative and quantitative components adequately	Are divergences and inconsistencies between quantitative and qualitative results adequately?	Different components of study adhere to the quality criteria of each tradition of methods involved?
van der Meij et al 2016	Y	Y	Y	Y	Y	Y	CT	NA	NA	Y	N	Y	N	Y	Y	Y	CT	Y	Y	Y	Y	Y	Y	N	N	N	N

Key

Green	Yes
Red	No
Yellow	Cannot tell
White	NA
